# Intelligence

**Published:** 1826-11

**Authors:** 


### INTELLIGENCE.

### MONTHLY REPORT OF PREVALENT DISEASES.

The close, hot, and damp weather, which has prevailed since our last report, has brought on a recurrence of those bowel-complaints which were so rife during the heats of summer. We mentioned last month that Fever had begun to make its appearance, and we hinted at the probability of the epidemic increasing: this accordingly has taken place, and fever, of a low typhoid character, is at present more prevalent in the metropolis than for some years past. The most remarkable feature in the disease is a tendency to assume, after a certain time, a remittent, or even intermittent type, and to run rather a lengthened course. We have seen no cases in which general blood-letting has been indicated; and, although symptoms of local determination have for the most part been present, yet the large proportion of those who have recovered under the use of free purging, and the application of leeches to parts which appeared to *labour*, has fully justified the conclusion that the treatment employed was not inefficacious. The present state of the atmosphere is favourable to the nurture of the fever, and it is therefore to be feared that we shall have future occasion to advert to the subject.

It has likewise fallen to the lot of the writer to see a considerable number of individuals, labouring under Pulmonary Consumption, terminate their lingering existence during the past month, justifying the common saying, that "they fall with the leaf." Although this has doubtless been in part accidental, yet almost every medical writer bears testimony to the greater number of deaths from this disease, which occur during the irregular weather of spring and autumn, than during the steady heat or cold of summer and winter.

The most interesting individual cases observed by the writer since last report, have been one of Tertian Ague, cut short by four grains of Sulphate of Quina; one of general Dropsy after Scarlatina, with epileptic fits, apparently from effusion into the brain, which was much benefited by general and local bleeding; and one of a *Blue Boy*, which, it is scarcely necessary to add, has not been much relieved by any treatment.

*October 25th.*

---

**Plagiarism from the London Medical and Physical Journal.*—We appeal to our readers, and to all our brother editors, whether we be not of a very peaceable disposition,—taking all the praise bestowed upon us with the utmost complacency, and bearing censure "with a patient shrug." Indeed, it is not a little gratifying to perceive how much some other Journalists find to praise, to condemn, or to extract, in the pages of the "Yellow Book." We confess, however, that there is one Journal which we think has used us ill,—it is the Edinburgh Journal of Medical Science. In the fourth and last Number of this are contained two papers,—one on Lithotomy, by Mr. Shaw, of the Middlesex Hospital; the other a remarkable case of Aneurism, which occurred to Mr. Charles Bell: the former is placed under the head of "Original Communications," and is said by the editor of the publication in question to be "*one of the most interesting in its details, and valuable in its consequences, that it has been at any time our duty to put on record.*"—Now, it will scarcely be credited that both these papers, as well as the engravings which accompany them, are taken, without acknowledgment, from the London Medical and Physical Journal.

So little were we disposed to suspect our northern contemporary of bad faith, that we at first believed (seeing that his "*original* department" was almost entirely made up of papers from other works,) that the reference had been accidentally omitted; but no exertion of charity could enable us to put this construction upon the matter, when we found a second paper, and a second engraving, in the same Number, likewise without any kind of acknowledgment. If it answers his purpose, let him say, "the London Medical and Physical Journal contains so

* As we perceive that the plagiarism is also mentioned in the advertisement of the present Number, inserted in the Newspapers, we take the opportunity of stating that all advertisements about the Journal come from the proprietors, not the—Editor.

much good matter, that we cannot do better than fill up our pages with its contents;" or, if he likes it better, let him say that the Journal is a bad one, and illustrate his position by extracting the communications of some of the most distinguished practitioners in the metropolis: in neither case should we complain. But do not let him talk of putting on record "most interesting" and "valuable" papers, which we have previously published. We trust that our brother editor will see the impolicy, not to say impropriety, of the line of conduct of which we complain: in which case, we beg respectfully to say that we have no bad feeling whatever towards him; but if not, we would just hint that we, too, come from the north of the Tweed, and have not forgotten our national motto.

---

*Letter from a Fellow of the College of Physicians, on the Jealousies of the Licentiates.*—Sir, As I perceive that the question respecting the Museum of the College of Surgeons, which was mentioned last month, for the second time in your Journal, is not yet understood; and as I am sure, from the uniform character of your observations on the College of Physicians, that you have no wish to promote dissention between the Fellows and Licentiates, I am induced to address you, in the hope that I may contribute towards clearing away what remains of misunderstanding on this subject.

It appears, in fact, that the whole discussion respecting the right of admitting visitors into the museum of the College of Surgeons, has arisen from a mistaken supposition that the trustees had the power of forming regulations respecting it, and had actually exercised that power in excluding the Licentiates from the privilege of introducing others, and allowing them only the right of personal admission.

Now, Sir, you will find on inquiry that, so far from this being the case, the trustees were appointed only to preserve the existing constitution of the museum, and not to legislate respecting it; and that all they did on this occasion was to promulgate the original regulation, or "Treasury minute," as it is called, which was delivered, with the museum, to the College of Surgeons, by the Lords of the Treasury.* And I think you will allow that, if we look at the conduct of the trustees with a due reference to the fact just stated, we shall have reason to say that those officers, so far from being blameable, deserve much credit for making public a regulation, valuable in itself, whatever defects it may have, which had lain completely dormant ever since the establishment of the museum.

Permit me to take this opportunity of referring also, in a few words, to the remarks of a most respectable quarterly Journalist, on the subject of the unkind feeling which is alleged to subsist amongst the Licentiates towards the Fellows of the College. There is, I assure you, no such disposition amongst the Fellows towards their brethren of the Scottish or foreign universities; nor can I see any reason for its prevalence amongst the Licentiates. It is true that, at the west end of the town, a large portion of the best medical practice has generally fallen to the share of the Fellows. But it can scarcely be supposed that the fellowship itself is the cause of this success; for the distinction between Fellow and Licentiate is not understood by the public. If the Fellows have any advantage amongst the higher classes, (I mean the gentry of England,) this evidently arises from their being, for the most part, educated with and early known to that class, and therefore, per-

* We beg to say that, had they taken the trouble to offer this explanation at once, we should not have thought it necessary to comment upon the subject at all. —A circumstance occurred at a meeting of the trustees which hurt the feelings of the Licentiates, (morbidly irritable, perhaps, in consequence of their exclusion from College privileges:)—it was suffered to go to the world without any explanation; and, although we stated the feelings of the profession upon the subject, and asked for explanation, (unwilling to believe the Censors capable of illiberality,) yet no notice was taken until the next Number of our Journal was completed; and even then the letter addressed to the editor was so deficient in perspicuity as to render its meaning ambiguous, while it reflected on him as requiring what no "liberal and intelligent person" would deem necessary. It must be acknowledged that we seldom meddle with medical politics, and it is probably this circumstance which made our remarks be so much more keenly felt than those of others. We shall only add, that, as we do not wantonly enter into such discussions, so they will find themselves mistaken who suppose that, having done so, we are to be deterred from our duty by any consideration whatever. (EDITOR.)

haps, being capable, on the whole, of a more ready assimilation to them in manner and feeling.

Besides, we may allow that there may be a disposition amongst the graduates of our own universities to assist one another, arising from those early associations which, in every profession in England, connects together individuals who have had the same education.

Now, if these be the true causes of the advantages which English graduates seem to enjoy, it is evident that they would still possess them if the College of Physicians were extinguished, or if every practising physician were called a Fellow of the College. What, then, is to be gained by perpetually charging on that body the effects of a distinction in truth quite independent of it?—a distinction which, I am disposed to think, would not excite any jealousy on the part of the Licentiates, if it were not for the qualification known to be necessary for its attainment: I mean an English education leading to an English degree.

We have often heard lately of the monopoly of the College of Physicians, but I am sure you have too much justice to promote so groundless a clamour. The College licenses the graduate of every university in Christendom who is found competent to practise, and places him, as to the privilege of acting as a physician, on an absolute equality with any of its Fellows. It only reserves, with some modifications to the graduates of the English universities, certain executive offices (which are miserably paid) in the English College of Physicians; a reservation which appears to me to be neither unreasonable in itself, nor calculated to give to the Fellow any advantage over the Licentiate in practice, independently of the circumstances above mentioned.

Whether any change might be advantageously made in the mode of electing Fellows, is a question which I will not enter upon here. But I can say with truth, that there is no want of liberal feeling amongst the Fellows of the College upon this or any other point touching the good of the profession. I am satisfied, however, that, should the fellowship cease to be generally a mark of an English degree, it would cease to be a desirable object with many of those who are now anxious to obtain it.

Allow me to trouble you, in conclusion, with a few words on the subject of the hostility which is said to exist between the Fellows and Licentiates, and to threaten some great disturbance in the profession. I cannot but deprecate such remarks as these, which appear to lead to no result whatever, except the excitement of useless jealousy between those who ought to live on good terms with each other. For I think it may be confidently said, that, *as long as the College of Physicians persists in the liberal system of admitting the graduates of every university in the world to practise physic, in all respects as freely as the Fellows, (after an examination similar to that which is submitted to by English graduates,) it has nothing to apprehend, even in this enlightened age, from the opposition of any body of men whatever.*

SOCIUS.

---

*List of New Officers of the College of Physicians.*—The following names have been added during the past year to the list of Fellows and Licentiates of the College of Physicians:

*Fellows.*—Dr. Thomas Watson, Henrietta-street, Cavendish-square; Dr. George Leith Roupell, Great Ormond-street; Dr. Richard Prichard Smith, Reading; and Dr. John Spurgin, Guildford-street.

*Licentiates.*—Dr. William Speir, Bartlett's Buildings; Dr. Samuel Millar; Dr. Thomas Hodgkin, New Broad-street; Dr. Richard Davie, Leamington; Dr. P. Frederick de Jersey; Dr. Æneas M'Andrew, Great Surry-street, Blackfriars; Dr. Charles Lush, Leadenhall-street; Dr. Francis Boott, Gower-street; Dr. John Wilton, Upminster; Dr. John Forbes, Chichester; Dr. George G. Sigmond, Dover-street; Dr. Charles Phillips, Union-street, Southwark; Dr. George B. Waddell, Charlotte-street, Blackfriars; Dr. Whitlock Nicholl, Old Burlington-street; Dr. Jas. Clark; Dr. Jas. Scott, Haslar Hospital; and Dr. C. Agar Hunt, Richmond.

*The Commissioners appointed under the "Act for Regulating Mad-houses."*—Sir H. Halford, Bart., Dr. Thompson, Dr. Yeats, Dr. Young, Dr. Macmichael, the Secretary, Dr. Bright, Curators of the Museum, the President, the Censors, Dr. Ager, Sir G. Tuthill, Dr. Paris, and Dr. Hawkins.

*Censors for the present year.*—Dr. Lambe, Dr. Cope, Dr. Southey, Dr. Hewett.

*Medical Provident Institution of Scotland.*—This institution deserves the attention of the profession, and we shall be happy to forward its objects in any way that may be in our power: with this view, we give insertion to the following official account of it.

The committee appointed by the meeting of 23d August (Professor Jameson, Dr. Morison, Dr. Beilby, Dr. Gairdner, Dr. Milligan, Dr. Wood, Dr Poole, Dr. Greville, Mr. Lizars, Dr. Campbell, Mr. George White, Dr. Spens, Dr. Borthwick, and Dr. Millar, of Edinburgh; Dr. Grace, and Dr. Mudie, of St. Andrew's; Dr. Spence, of Cupar; Dr. Walter Graham, of Dalkeith; and Mr. Allison, of Leith,) having met, and deliberated upon the description of benefits to be granted by this institution, were of opinion that it should embrace all those specified in a paper formerly printed and circulated among the members of the profession, under the title of "Hints with a view to the Formation of a Society among the Medical Profession of Scotland;" but that it shall be optional to take any one or more of them. These benefits are as follows:

1. Annuities payable weekly or monthly during professional incapacity, succeeded by annuities certain in old age. 2. Annuities in old age, unconnected with any benefit during professional incapacity. 3. The assurance of sums payable at death, but convertible at the option of the assured into annuities payable during their own lives, or the lives of their widows or nominees. 4. Annuities to widows or nominees, unconnected with life assurance. 5. Endowments for children in the form of a tontine;—the accumulated fund of each class to be divided among the survivors at different ages, not exceeding that of twenty-one, so as to provide for their education, and setting out in the world.

The committee have also had under their consideration the expediency of receiving small sums from their members, to be accumulated at compound interest, and the whole proceeds returned to them after a certain period.

The institution would thus unite in their scheme all the benefits now obtainable from, 1st, Friendly Societies; 2d, Life Assurance Companies; and 3d, Saving Banks. It has also been suggested that members might be accommodated with certain sums on loan, on security of their previous payments to the funds of the institution.

These several departments of business will be kept entirely distinct, and any surplus that may accrue on any one of the funds will be exclusively appropriated to the benefit of those who have contributed to such fund; while there will be a great comparative saving in the expence of management, by combining all these branches in one scheme.

Some of these benefits, it is well known, are not attainable at present from any existing institution; and others, such as life assurance and widows' annuities, have not hitherto been presented to the public in so beneficial a form. A person who makes an assurance on his own life, for instance, with the present companies, has no other choice than to continue his annual payments till death, or lose entirely, or in a great measure, all that he has already paid: he must either forfeit his policy, or dispose of it at a great disadvantage. In this institution, on the other hand, he will have the option, after attaining a certain age, of converting the full value of his policy into one or other of the following annuities:—1, An annuity for the rest of his own life; or, 2, an annuity for the joint lives of himself and wife, or other nominee, and for the life of the survivor; or, 3, an annuity for his widow or nominee.

As there will be no body of proprietors distinct from the members, the whole funds will be shared among the latter, according to their respective rights and interest; while they will incur no responsibility beyond the sums they have agreed to contribute.

As a few weeks must elapse before the necessary tables of payments and benefits can be prepared, the following approximations, and which are to be considered merely as such, are submitted in the mean time.

An yearly payment of *two pounds twelve shillings*, commencing at the age of 21, and continued till 60, will entitle to an annuity during professional incapacity, at the rate of *one pound* per week till 60, and an annuity certain of *twenty-six pounds* for the remainder of life; and an annual payment of *five guineas* will purchase the right to double these sums, or *two pounds* per week during professional incapacity, and an annuity of *fifty-two pounds* for life after 60. Thus, for a payment not exceeding *one shilling* per week in the one case, and about *two shillings* in the other, a member entering at the age of 21 will be assured against the privations of sickness, infirmity, and old age, through the remainder of life. Those who enter at a higher age than 21, will of course be required to make a corresponding increase in the annual payment.

Again, a member assuring 1000*l.* upon his life at the age of 25, at an annual premium of about *two guineas per cent.*, will have the option either to continue his payments till death, and leave this sum to be paid then, in any way he may think proper; or, at the age of 60, he may discontinue his payments, and convert his policy into one or other of the following annuities:—1, An annuity of *thirty-five pounds* for the remainder of his own life, payable half-yearly, and down to the moment of death; or, 2, an annuity of *twenty-six pounds* during the joint life of himself and wife, or other nominee, five years younger than himself, and for the life of the survivor, and payable as above; or, 3, an annuity of *seventy-five pounds* for the life of his widow, or nominee; the difference of age and the payment of the annuity being as before.

But, with a view to the expence necessarily incurred at death, a full year's annuity will be paid to the representatives of the deceased, over and above what may be due at the termination of the life, whenever that may happen.

All the benefits dependent upon life or health may be purchased either by one single payment, or by an annual payment continued for seven, fourteen, or twenty-one years only.

The committee, in submitting this statement to the members of the profession, do not feel it necessary at present to enter into further details. The scheme, in all its parts, requires only to be understood to recommend itself to their support and encouragement, whatever may be the rank they hold, and whether they do or do not mean themselves to participate in its peculiar advantages.

Agents will be appointed throughout the country, from whom, in a short time, information regarding the necessary tables and arrangements may be received, and to whom communications and proposals may be addressed.

*Edinburgh; 23d August,* 1826. JAS. CLEGHORN, *Secretary.*

---

*Literary Notice.*—Mr. William Barrett Marshall, Assistant Surgeon R.N., Honorary M.D. of the University of Gottingen, and Author of "Tears for Pity," proposes to publish, (as soon as a sufficient number of subscribers have been obtained to defray the expenses of publication,) in demy 12mo. price 5s. "An Essay on Medical Education, in which the relative Importance of the various accessory Sciences is ascertained, and the extent to which each separate Science, or Branch of general Knowledge, should be cultivated, to afford the highest degree of Usefulness in the Healing Art. Dedicated, by permission, to the Medical Commissioners of His Majesty's Navy."—To the original Essay, the Associated Apothecaries and Surgeon-Apothecaries of England and Wales awarded their gold medal, on the 7th of July, 1824.

### METEOROLOGICAL JOURNAL,

*From September 20th, to October 20th, 1826.*

By Messrs. HARRIS and Co. Mathematical Instrument Makers, 50, High Holborn.

| Sept. | Moon. | Rain gauge. | Thermom. 9 A.M. | Thermom. MAX. | Thermom. MIN. | Barometer. 9 A.M. | Barometer. 10 P.M. | De Luc's Hygrom. 9 A.M. | De Luc's Hygrom. 10 P.M. | Winds. 9 A.M. | Winds. 10 P.M. | Atmospheric Variations. 9 a.m. | Atmospheric Variations. 2 p.m. | Atmospheric Variations. 10 p.m. |
|---|---|---|---|---|---|---|---|---|---|---|---|---|---|---|
| 20 | | | 60 | 64 | 52 | 29.76 | 29.86 | 92 | 75 | NE | NE | Rain | Fair | Fair |
| 21 | | | 57 | 64 | 46 | 29.96 | 30.03 | 74 | 71 | NE | NE | Fair | Fine | — |
| 22 | | | 48 | 60 | 44 | 30.07 | 30.04 | 74 | 71 | SE | E | — | — | Fine |
| 23 | | | 51 | 61 | 52 | 29.97 | 29.86 | 75 | 74 | ESE | E | Fine | — | Cloudy |
| 24 | ☾ | | 54 | 64 | 59 | 29.70 | 29.63 | 85 | 89 | ESE | SSE | Cloudy | Cloudy | Rain |
| 25 | | | 61 | 65 | 52 | 29.62 | 29.70 | 91 | 85 | SE | SW | Clo. Ra. | — | Cloudy |
| 26 | | .70 | 62 | 66 | 59 | 29.76 | 29.84 | 90 | 90 | SW | SSW | Rain | Fair | Rain |
| 27 | | | 63 | 68 | 58 | 29.87 | 29.97 | 77 | 84 | W | SW | Cloudy | — | Fair |
| 28 | | | 65 | 69 | 55 | 30.05 | 30.09 | 83 | 85 | SW | SW | Fair | — | — |
| 29 | | | 58 | 69 | 65 | 30.04 | 29.84 | 88 | 84 | SW | ESE | — | — | — |
| 30 | | | 65 | 70 | 51 | 29.67 | 29.73 | 87 | 85 | SSW | SW | — | Cloudy | — |
| Oct. | | | | | | | | | | | | | | |
| 1 | ● | | 57 | 65 | 47 | 29.73 | 29.79 | 85 | 81 | SW | WSW | — | Sm. Ra. | Fine |
| [illegible] | | | 49 | 61 | 48 | 29.84 | 29.85 | 87 | 80 | SW | WSW | Foggy | Fair | Cloudy |
| 3 | | | 50 | 60 | 49 | 29.81 | 29.80 | 84 | 87 | WSW | W | Fair | — | Fair |
| 4 | | | 50 | 58 | 42 | 29.66 | 29.68 | 85 | 74 | WSW | N | Foggy | — | Fine |
| 5 | | | 44 | 54 | 39 | 29.67 | 29.82 | 78 | 71 | W | NNW | — | Fine | — |
| 6 | | | 41 | 53 | 41 | 29.98 | 30.07 | 75 | 68 | NNW | W | — | — | Foggy |
| 7 | | | 44 | 59 | 42 | 30.00 | 29.93 | 75 | 78 | W | SSW | — | Fair | Fair |
| 8 | ☽ | .38 | 58 | 62 | 48 | 29.83 | 29.70 | 86 | 86 | SW | WSW | Cloudy | Rain | Rain |
| 9 | | | 51 | 57 | 46 | 29.69 | 29.70 | 82 | 78 | SW | WSW | — | Fair | Fine |
| 10 | | | 50 | 67 | 58 | 29.63 | 29.72 | 89 | 89 | SSW | W | Rain | R a | Cloudy |
| 11 | | .25 | 61 | 67 | 59 | 29.92 | 30.00 | 89 | 84 | WSW | WSW | Foggy | R.&Fair | — |
| 12 | | | 61 | 65 | 59 | 29.99 | 29.96 | 92 | 81 | WSW | SW | Rain | Fair | — |
| 13 | | | 61 | 67 | 47 | 29.93 | 30.10 | 89 | 73 | WSW | N | Fair | — | Fine |
| 14 | | | 49 | 60 | 55 | 30.11 | 30.11 | 76 | 85 | NE | E | — | — | — |
| 15 | ○ | | 60 | 65 | 55 | 29.82 | 29.62 | 86 | 86 | SE | SSE | — | — | — |
| 16 | | .15 | 58 | 65 | 45 | 29.54 | 29.66 | 90 | 76 | S | WSW | — | Rain | — |
| 17 | | | 46 | 60 | 50 | 29.89 | 29.88 | 81 | 79 | SSW | E | Foggy | Fair | — |
| 18 | | | 55 | 61 | 57 | 29.87 | 29.91 | 93 | 88 | E | ENE | — | — | Cloudy |
| 19 | | | 60 | 66 | 58 | 29.89 | 29.89 | 92 | 95 | E | E | Cloudy | — | — |

The quantity of Rain fallen in the month of September, was 2 inches and 99.100ths.

